# Method of Failure Diagnostics to Linear Rolling Guides in Handling Machines

**DOI:** 10.3390/s23073770

**Published:** 2023-04-06

**Authors:** Radka Jírová, Lubomír Pešík, Lucia Žuľová, Robert Grega

**Affiliations:** 1 Faculty of Mechanical Engineering, Technical University of Liberec, Studentska 2, 461 17 Liberec, Czech Republic; 2 Faculty of Mechanical Engineering, Technical University of Košice, Letna 9, 042 00 Kosice, Slovakia

**Keywords:** failure diagnostics, damage diagnostics, vibrations, acceleration measurement, linear rolling system

## Abstract

Linear rolling guides, used in production machines for the realisation of linear motion, demand in industrial practice early damage identification to prevent production outages and losses. Therefore, the article aims for early damage diagnostics that use the principle of a load-free diagnostic part integrated into the carriage of the linear rolling guide. This principle was employed for developing an innovative method of damage identification to a guiding profile or rolling elements. The proposed innovative method is based on analysing vibration acceleration measured on the diagnostic part in the context of carriage position. In addition, a unique connection of an acceleration sensor to the diagnostic part through a mechanical component with defined parameters of stiffness and mass was designed. The innovative method was verified by laboratory testing on a designed functional sample of the diagnostic system. The computed reliability of the proposed diagnostic method reached 98%.

## 1. Introduction

Linear rolling guides are widely used in industrial practice as a part of production lines and production machines for realising a relative linear motion. Linear motion mechanisms are often used in CNC machines, industrial robots, or handling machines, wherein they ensure the primary function of these machines—positioning or transporting objects. As linear rolling guides are basic components of linear mechanisms, their failure means disabling machine function, stopping the production process, and, consequently, generating production losses [1,2]. Therefore, production companies expect high reliability and demand prediction of possible failures.

In linear rolling guides, the failure occurs as “pitting”, which means breaking out surface particles of rolling elements or the guiding profile [3,4,5]. This process of fatigue failure finally leads to the breakage of the carriage end-cover and to the loosening of rolling elements. The bases of damage progression define a wide range of physical principles and diagnostic methods that are currently used for wear identification in linear rolling guides. These principles and methods follow up on knowledge of rolling bearing diagnostics, which is mainly based on vibration, noise, or rolling resistance measurements [6,7,8] in the context of various types of signal processing [9,10,11].

Nevertheless, significant differences can be observed between the kinematic and dynamic behaviour of linear rolling guides and rolling bearings. One of the most important is the difference between linear and rotary motion. While linear motion varies throughout the movement by accelerating and decelerating, rotary motion is mostly constant or partially constant through operation cycles. So, methods based on frequency analysis are not applicable to wear identification in linear rolling guides [12]. The second limitation is connected with load transmission through the linear and rotary rolling systems. In rolling bearings, theoretically, one rolling element at a specific time transmits the external load between the raceway of an inner and outer ring. When damage occurs, the fully loaded rolling element, by crossing over the damage, excites vibration and noise signals, and generates increased rolling resistance. Therefore, early damage in the rolling bearing can be detected. In linear rolling guides, the load is transmitted through a row of rolling elements. Therefore, due to the initial damage, the adjacent rolling elements take over the load, and a signal is not triggered by crossing one rolling element over the damage.

Thus, current diagnostic methods, which are further described, mainly enable damage identification at the later stage of progression. This generates uncertainty in industrial practice about whether the damage is detected in sufficient time to prevent production outages and losses. The examples of greatly damaged linear rolling guides without any damage signalisation of the diagnostic used are well-known in industrial practice [13].

One of the current principles regards the measurement of end-cover deflection, which relates to the increased inner pressure on the end-cover raceway due to increased rolling resistance by failure. The producer, Schaeffler (Schaeffler Technologies AG & Co. KG, Herzogenaurach, Germany), designed a free deformable part in the end-cover for evaluating functional status [14]. Development of this principle focuses on its material properties to ensure strength and elasticity and, thus, sufficient sensitivity of the failure diagnostics [15,16,17].

Next, researchers and producers have focused on developing diagnostic systems based on progressive analysis of measured acceleration of vibrations through, e.g., neural networks or other advanced algorithms. These methods are related to the lubrication level, as Feng [18] explained that lubrication of linear rolling guide influences root mean square (RMS) values of measured vibrations and their distribution in the frequency domain. A patent [19] of Japanese producer THK (THK Co., Ltd., Tokyo, Japan) and a patent application [20] of the producer Schaeffler use proposed recognition to diagnose linear rolling guides.

In the context of increasing the reliability of damage identification, researchers [21,22,23,24] still endeavour to find more appropriate entrance parameters and a more suitable neural networks algorithm for the status evaluation of linear rolling guides. There are efforts to compare different types of vibrational signal analysis, such as RMS, spectral analysis, crest factor, or spectrogram.

According to the insufficient reliability of current diagnostic systems, the producer Škoda (Škoda Auto a.s., Mlada Boleslav, Czech Republic), in cooperation with the TUL (Technical University of Liberec, Liberec, Czech Republic) scientific team, has introduced an original principle of linear rolling guides diagnostics [25]. The principle aims for early failure diagnostics even if the linear rolling guides are operated under great external loads. The diagnostics dispose of a load-free diagnostic part integrated into a linear guide carriage. Reflecting on the initial testing [13], this original principle seems to be a progressive method for status assessment, which is independent of operating conditions of linear rolling guides and may provide failure identification in a real-life situation soon.

Therefore, the article focuses on developing an evaluation method for early failure identification utilising the diagnostic part. The proposed innovative diagnostic method is based on evaluating vibrations measured on the diagnostic part in the context of the carriage position on the guiding profile. It can be noticed by processing measured signals in the time domain that increased peaks of vibrations related to the damage can be observed at the same carriage position by forward and backward movement. The innovative diagnostic method employs a unique connection of a vibration sensor to the diagnostic part. The unique connection is realised through mechanical parts with defined dynamic parameters of stiffness and mass. Thus, the frequency of vibrations related to the damage can be tuned outside the machine’s operating frequencies. The combination of processing measured signals at the time domain in the context of carriage position and the unique sensor connection led to the high reliability of early damage identification proved in laboratory conditions.

The structure of the article consists of four sections: Materials and Methods, Theoretical Background, Results and Discussion, and Conclusions. In the section on Material and Methods, the article describes the innovative method of damage identification and the methodology of experimental evaluation of the proposed method in laboratory conditions. The section Theoretical Background clarifies the mechanical principles used for developing the innovative method for damage identification. In the section Results and Discussion, the results of experimental tests are summarised and discussed through a statistical evaluation of the proposed diagnostic method reliability. In Conclusions, the main benefits of the innovative diagnostic method are pointed out.

## 2. Materials and Methods

**Figure 1:**
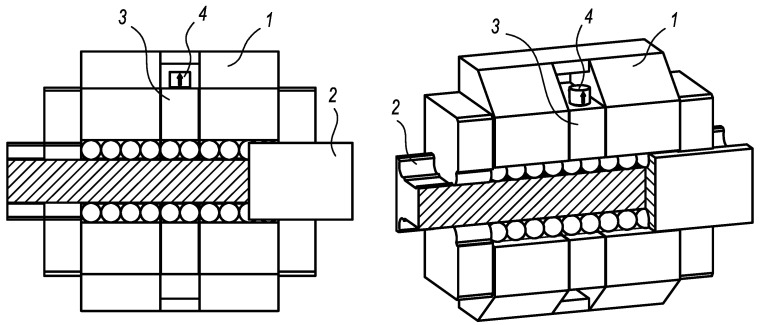
The linear rolling guide with the integrated diagnostic part: 1—carriage, 2—guiding profile, 3—diagnostic part, and 4—acceleration sensor.

The presented diagnostic system (Figure 1) uses an integrated diagnostic part with an acceleration sensor for vibration measurement [25]. The design of the diagnostic part has to fulfil basic requirements, such as minimal weight, load-free status, and minimal length, given generally by two diameters of rolling elements and sharing rolling elements and the guiding profile with the loaded carriage. Owing to the load-free status of the diagnostic part, the independence of various operating and external conditions is ensured. For achieving high sensitivity of diagnostic function, the length of the diagnostic part should be as small as possible. So, the length requirement of two diameters of rolling elements is related to the functional stability during the operation. The acceleration sensor is placed on the diagnostic part to identify vibrations caused by the damage of rolling elements or the guiding profile.

Utilising this original diagnostic principle, an innovative method of status assessment of linear rolling guide has been developed. The method requires putting the measured vibration data in the context of distance, which is realised by the linear rolling guide. Then, the decision of whether increased peaks of vibrations relate to the damage may be reached. The damage is detected when the increased peaks occur at the same position by the forward and backward movements. Thus, the solution demands measurement of distance that may be realised by, e.g., presence sensor or, in the presented case, by the output data from the electromotor.

Next, the proposed method applies innovative attachment of the acceleration sensor. Standard attachment is provided by an almost rigid connection through a magnet, a thread, or an adhesive [26,27]. The unique connection brings a definition of the stiffness and additional mass properties of the sensor. By defining these dominant parameters of the sensor connection, the frequency of vibrations related to the damage can be shifted into the band out of the operating frequencies of the machine.

The design of the diagnostic part uses a small preload to delimit clearances between the diagnostic part, the rolling elements, and the guiding profile.

The proposed diagnostic method was verified via laboratory testing on a functional sample. The testing process focused at first on comparing the standard and unique connection of the acceleration sensor. After that, laboratory tests pointed to verification of the proposed method in the case of the rolling elements and guiding profile damage.

**Figure 2:**
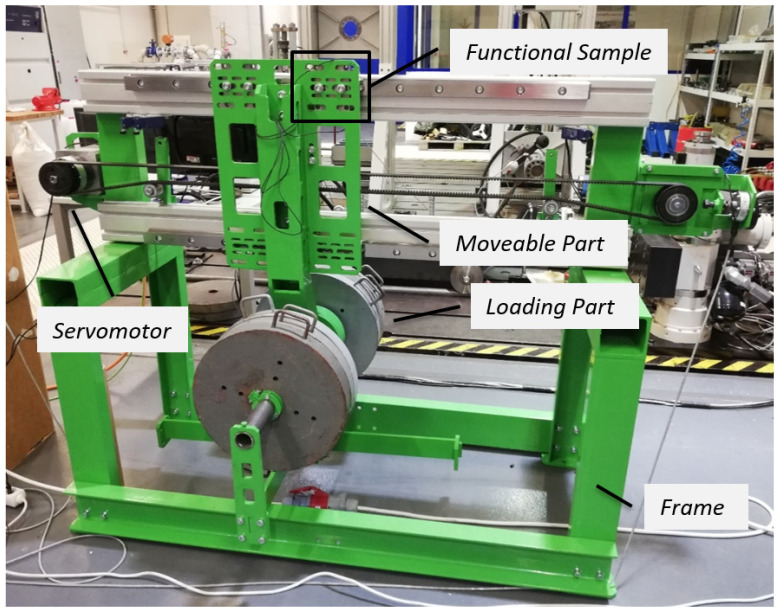
Test setup.

The designed testing facility enabled the simulation of variable operating conditions of linear rolling guides, specifically, the loading of the functional sample via a set of weights and the definition of kinematic properties for a linear motion. The testing facility consists of a frame, a moveable part, and a loading part (Figure 2). The moveable part holds and moves the functional sample with the integrated diagnostic part along the guiding profile, which is connected to the frame. Next, the loading part with the set of weights is linked to the moveable part. The linear motion was ensured by a servomotor through a toothed belt, which provides a proper drive without undesirable vibrations. During the testing process, the linear motion was defined by an acceleration of *a* = 1 ms^−2^, a velocity of *v* = 0.4 ms^−1^, and a distance of s = 0.8 m. The functional sample was loaded with weights of *m* = 300 kg.

**Figure 3:**
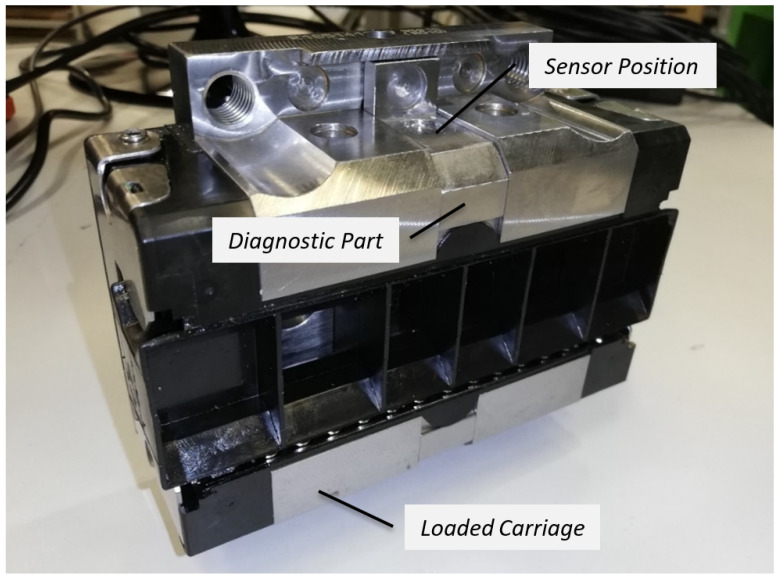
The functional sample with the integrated diagnostic part.

The functional sample with the integrated diagnostic part was produced utilising the Bosch Rexroth (Bosch Rexroth AG, Lohr am Main, Germany) linear rolling guide. The carriage with parameters summarised in Table 1 was redesigned according to Figure 3. Respecting the diameter of rolling elements, the length of the diagnostic part was designed to be 18 mm. The functional sample enabled the placing of the acceleration sensor through the standard and unique connection.

The 1-axis acceleration sensor of the producer MMF (Manfred Weber Metra Meß- und Frequenztechnik in Radebeul e.K., Radebeul, Germany) was employed for evaluating the vibrations on the diagnostic part. The acceleration sensor, type KS97.100, featured a range of ±60 G and a linear frequency range of up to 10 kHz. For the complex status evaluation of the linear rolling guide, the servomotor provided time data for the distance travelled. A sampling frequency of both (vibrations and distance) measurements was set to 200 kHz. Measured data of vibration acceleration, analysed at the time domain, was filtered by a low pass FIR (finite impulse response) filtering with a cut-off frequency of 9 kHz, transition band of 100 Hz, and Hanning window type. In addition, the frequency spectrum of measured vibrations was computed utilising the Hanning window type and resolution of 10 Hz. Measurements and data analyses were processed utilising DEWESoftX.

The testing process was realised with a confidence level of 95%. Measurement uncertainty of the measurement chain, including the acceleration sensor, was determined at 1000 Hz frequency and a reference value of acceleration 100 ms^−2^. However, it can be assumed that a similar measurement uncertainty would be reached as well for other frequencies. The obtained result was 100.1 ± 1.4 ms^−2^ with a probability of 95%.

**Figure 4:**
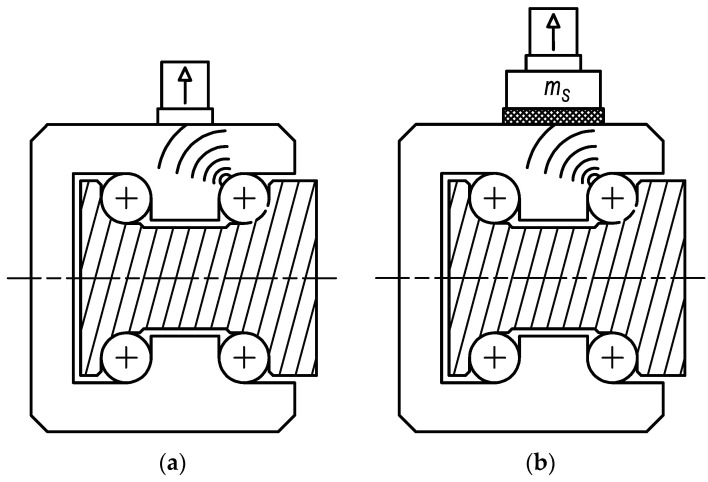
The scheme of sensor connection: (**a**) with magnet; and (**b**) with rubber spring and additional weight.

The first stage of testing focused on verifying the proposed status assessment method in the context of the unique connection applied for the acceleration sensor. Figure 4 displays the scheme of standard and innovative connection of the sensor to the diagnostic part. The proposed connection method is based on utilising a rubber spring and additional weight to define the optimal frequency of vibrations related to the damage. The rubber spring of microporous EPDM (ethylene propylene diene monomer) material, with a thickness of 3 mm and Shore hardness of 125 A, was used in the tests. A mass of additional weight was 14 g. The standard sensor connection was provided by a magnet.

**Figure 5:**
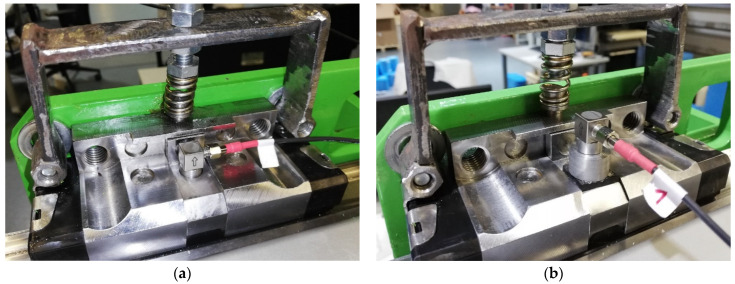
The placing of acceleration sensor: (**a**) with magnet; and (**b**) with rubber spring and additional weight.

Figure 5 shows the actual sensor position in the case of connection with the magnet and the rubber spring with additional weight.

**Figure 6:**
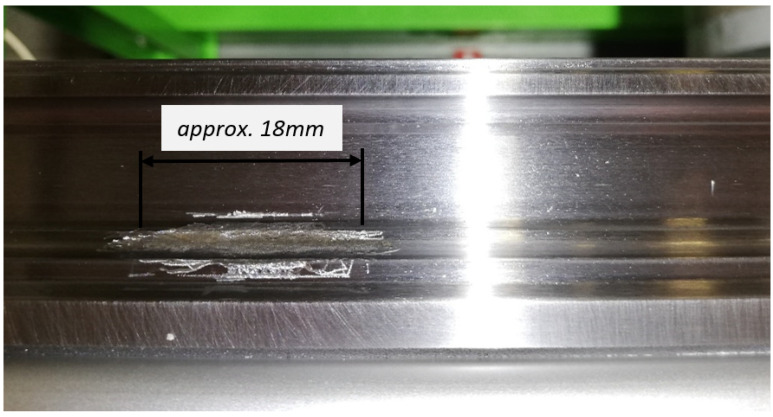
The simulated damage on the guiding profile.

The first stage measurements were performed at the simulated damage on the guiding profile as a ground groove (Figure 6), which substituted a pitting fatigue failure. The ground groove was a depth of approx. 0.1 mm and a thickness of a limit range of damage, which is related to the length of the diagnostic part of 18 mm.

The second stage of testing verified the proposed status assessment method in the simulation of rolling elements damage. The rolling elements damage was simulated utilising four adjacent elements with the diameter of 7.9 mm, so 0.1 mm less than the diameter of the original balls.

The complexity of the testing process was reached by methodical procedure:

- Laboratory facility visual check;
- Lubrication of the diagnostic part with oil;
- Initial running of testing facility for 30 forward and 30 backward movements at defined kinematic parameters;
- The testing cycle of 30 forward and 30 backward movements at defined kinematic parameters.

Finally, the results of the testing process were statistically evaluated as the reliability of damage identification by one forward and backward movement.

## 3. Theoretical Foundation

**Figure 7:**
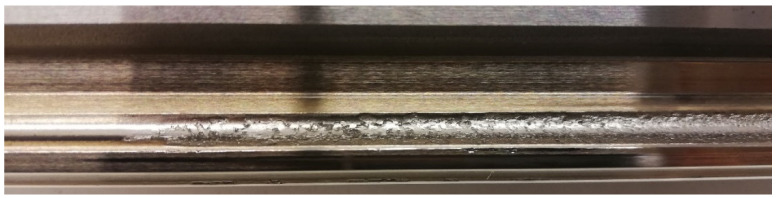
The pitting of the guiding profile.

When in service, linear rolling guides are operated under external loads that cause a contact pressure between rolling elements and the guiding profile. Further, the linear motion is characterised by a rolling of the rolling element over the guiding profile. It leads to the cyclic loading and unloading of contact surfaces resulting in the dynamical character of contact pressure. The dynamic changes of contact pressure may generate a fatigue failure, so-called “pitting” or “spalling”, caused by insufficient lubrication [28,29,30]. The result of pitting fatigue failure is the breakout of contact surface particles and generation of pits (Figure 7). By insufficient lubrication in the service, the surface layer is spalled as a result of fatigue failure.

The progression of fatigue failure may lead to the components’ destruction of linear rolling guides. The consequence of fatigue wear is an increment in vibrations of the linear rolling guide [31,32]. These vibrations create, in general, two excitation principles, force and kinematic, of adjacent components, which constitute a dynamical system.

Force excitation relates to the transition of rolling elements from a non-loaded to a loaded state that acts as an inner shock force between the carriage and the rolling element and between the rolling element and the guiding profile. This process typically occurs in service of linear rolling guides without any damage due to the recirculation of rolling elements in the non-loaded and loaded raceway of the carriage [33]. Respecting the fact that the character of excited vibrations is similar to the guiding profile damage, the force excitation cannot be decisive in the status identification of linear rolling guides.

The damage of the linear rolling guide is more intensive in the way of the kinematic excitation when the vibrations are generated and transmitted to the carriage or the diagnostic part through movement over the damaged contact surfaces. This process is applied in the proposed status assessment of linear rolling guides.

**Figure 8:**
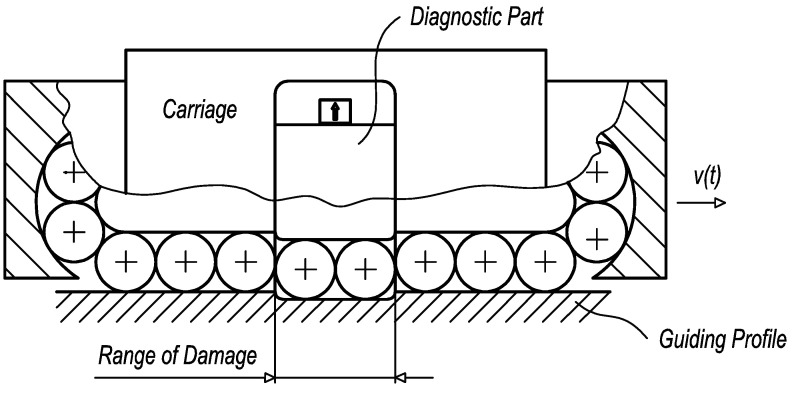
The limit range of guiding profile damage.

In practice, the design of the diagnostic part and its dimensions affect the creation of kinematic excitation. Two primary cases when kinematic excitation appears may be described. In the case of the guiding profile damage, the limit range of the damaged surface has to be greater than the length of the diagnostic part (Figure 8).

**Figure 9:**
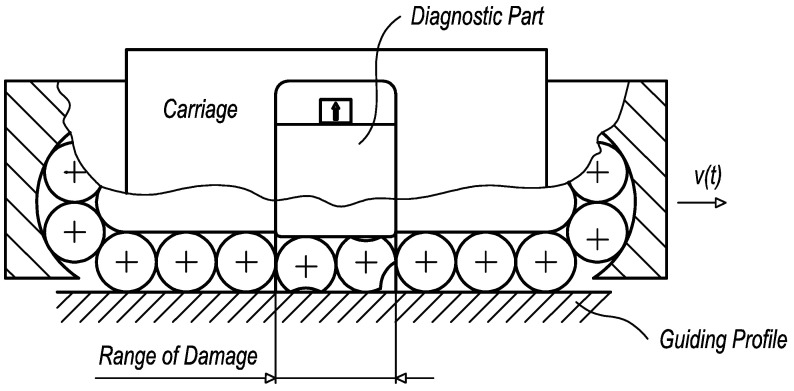
The limit range of rolling elements damage.

In the case of the rolling elements damage, the limit range is related to the adjacent rolling elements which fill the overall length of the diagnostic part (Figure 9).

**Figure 10:**
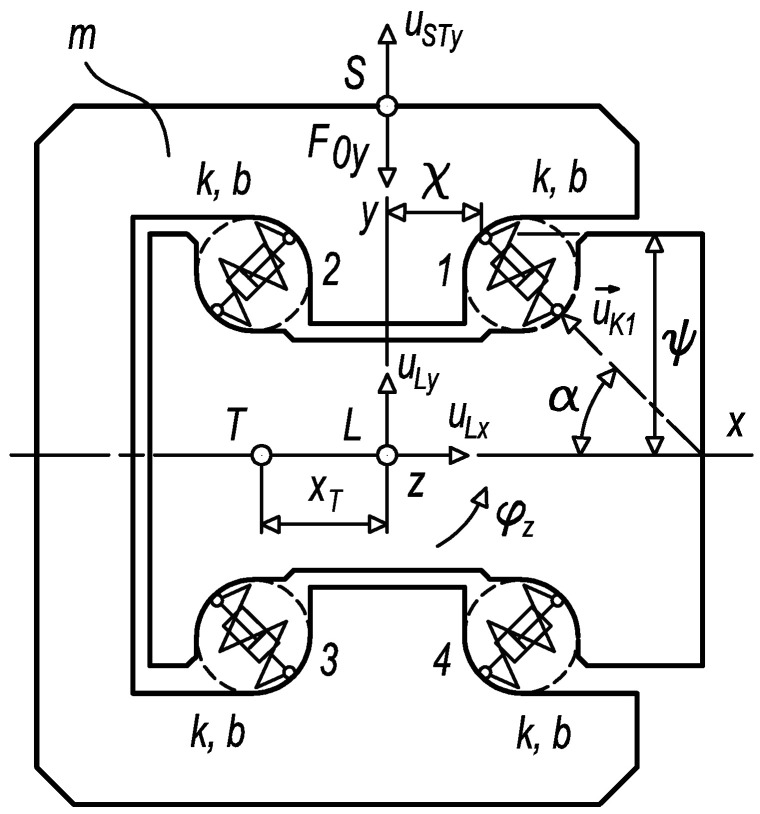
The mechanical model of the diagnostic part.

The response of the diagnostic part to the kinematic excitation at the limit range of damage can be solved through a mechanical model depicted in Figure 10 [34,35]. The mechanical model represents a dynamic system of the diagnostic part, where elastic and damping links substitute rolling elements and mass properties interpret a body of the diagnostic part. A simplified mechanical model applies general planar motion; sliding connections between the diagnostic part and the loaded carriage are not considered.

The dynamic system, according to Figure 10, is preloaded by a small force of *F*_0*y*_ equal to 10 N. The value of the stiffness k used reflects the stiffness of two rolling elements in contact with the raceway of the diagnostic part. The damping coefficient b of the dynamic system (1) approximately equals:

(1) $$b≅2b_{rel}\sqrt{4km}$$

wherein *b_rel_* is a damping ratio with a value of 0.05 [36]. The overall mass properties and parameters of elastic and damping links are specified in Table 2 and Table 3.

**Figure 11:**
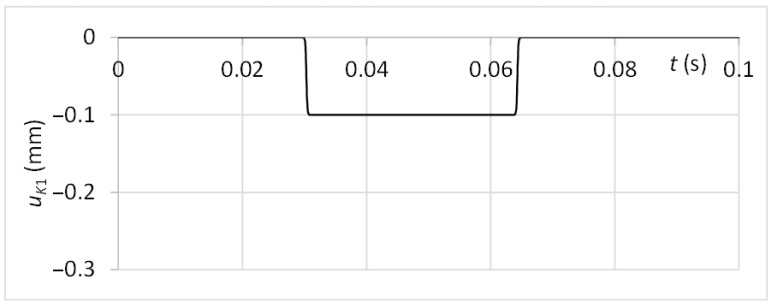
The kinematic excitation of the diagnostic part.

The system is excited by a function *u_K_*_1_ with the character respecting the linear motion velocity of 0.4 ms^−1^ and the guiding profile damage with a thickness of the limit range and depth of 0.1 mm. The time graph of the kinematic excitation function is shown in Figure 11.

Through the differential motion equations, the vibration motion of the diagnostic part at the acceleration sensor position S may be computed. The differential motion equations can be constructed through the Lagrange Equation (2) in the vector form [36,37].

(2) $$\frac{d}{dt}\left(\frac{\partial E}{\partial \vec{\dot{q}}}\right)+\frac{\partial U}{\partial \vec{q}}+\frac{\partial D}{\partial \vec{\dot{q}}}=\vec{F}$$

wherein *E* is kinetic energy, *U* is potential energy, *D* is dissipative energy, $\vec{F}$ is generalised force and moment, $\vec{q}$ is generalised position, and $\vec{\dot{q}}$ is generalised velocity. As generalised position resp. velocity are submitted into Equation (2) vector of position ${\vec{u}}_{L}$ resp. velocity ${\vec{\dot{u}}}_{L}$ and vector of angular position $\vec{\varphi }$ resp. velocity $\vec{\dot{\varphi }}$.

The first term of Equation (2) for the generalised velocity $\vec{\dot{q}}$ represented by the vector of velocity ${\vec{\dot{u}}}_{L}$ (3) and the vector of angular velocity $\vec{\dot{\varphi }}$ (4) becomes:

(3) $$\frac{d}{dt}\left(\frac{\partial E}{\partial {\vec{\dot{u}}}_{L}}\right)=m{\vec{\ddot{u}}}_{L}-S_{L}\vec{\ddot{\varphi }}$$

(4) $$\frac{d}{dt}\left(\frac{\partial E}{\partial \vec{\dot{\varphi }}}\right)=S_{L}{\vec{\ddot{u}}}_{L}+J_{L}\vec{\ddot{\varphi }}$$

wherein $S_{L}$ represents the antisymmetric tensor (5) related to the vector of the first moment of inertia respecting the origin *L* of the local coordinate system and $J_{L}$ is the tensor of inertia (6) respecting the origin *L* of the local coordinate system.

(5) $$S_{L}=\left[\begin{array}{ccc} 0 & 0 & 0\\ 0 & 0 & -x_{T}m\\ 0 & x_{T}m & 0 \end{array}\right]$$

(6) $$J_{L}=\left[\begin{array}{ccc} J_{x} & 0 & 0\\ 0 & J_{y} & 0\\ 0 & 0 & J_{z} \end{array}\right]$$

The second term of Equation (2) for the generalised position $\vec{q}$ represented by the vector of position ${\vec{u}}_{L}$ (7) and the vector of angular position $\vec{\varphi }$ (8) is:

(7) $$\frac{\partial U}{\partial {\vec{u}}_{L}}=k\overset{n}{\underset{i=1}{\sum }}\lambda_{i}\left({\vec{u}}_{L}-P_{i}\vec{\varphi }-{\vec{u}}_{Ki}\right)$$

(8) $$\frac{\partial U}{\partial \vec{\varphi }}=k\overset{n}{\underset{i=1}{\sum }}P_{i}\lambda_{i}\left({\vec{u}}_{L}-P_{i}\vec{\varphi }-{\vec{u}}_{Ki}\right)$$

The third term of Equation (2) for the generalised velocity $\vec{\dot{q}}$ represented by the vector of velocity ${\vec{\dot{u}}}_{L}$ (9) and the vector of angular velocity $\vec{\dot{\varphi }}$ (10) equals:

(9) $$\frac{\partial D}{\partial {\vec{\dot{u}}}_{L}}=b\overset{n}{\underset{i=1}{\sum }}\lambda_{i}\left({\vec{\dot{u}}}_{L}-P_{i}\vec{\dot{\varphi }}-{\vec{\dot{u}}}_{Ki}\right)$$

(10) $$\frac{\partial D}{\partial \vec{\dot{\varphi }}}=b\overset{n}{\underset{i=1}{\sum }}P_{i}\lambda_{i}\left({\vec{\dot{u}}}_{L}-P_{i}\vec{\dot{\varphi }}-{\vec{\dot{u}}}_{Ki}\right)$$

wherein $P_{i}$ signs the antisymmetric tensor related to the vector of the position of the *i*-th elastic and damping link. The antisymmetric tensors $P_{1}$ to $P_{4}$ of elastic and damping link 1 to 4 (11)–(14) equal:

(11) $$P_{1}=\left[\begin{array}{ccc} 0 & 0 & \psi \\ 0 & 0 & -\chi \\ -\psi & \chi & 0 \end{array}\right]$$

(12) $$P_{2}=\left[\begin{array}{ccc} 0 & 0 & \psi \\ 0 & 0 & \chi \\ -\psi & -\chi & 0 \end{array}\right]$$

(13) $$P_{3}=\left[\begin{array}{ccc} 0 & 0 & -\psi \\ 0 & 0 & \chi \\ \psi & -\chi & 0 \end{array}\right]$$

(14) $$P_{4}=\left[\begin{array}{ccc} 0 & 0 & -\psi \\ 0 & 0 & -\chi \\ \psi & \chi & 0 \end{array}\right]$$

The tensor $\lambda_{i}$ is related to the unit direction vector of the *i*-th elastic and damping link. The tensors $\lambda_{1}$ to $\lambda_{4}$ of elastic and damping link 1 to 4 (15), (16) equal:

(15) $$\lambda_{1}=\lambda_{3}=\left[\begin{array}{ccc} \cos^{2}\alpha & -\cos\alpha \sin\alpha & 0\\ -\cos\alpha \sin\alpha & \sin^{2}\alpha & 0\\ 0 & 0 & 0 \end{array}\right]$$

(16) $$\lambda_{2}=\lambda_{4}=\left[\begin{array}{ccc} \cos^{2}\alpha & \cos\alpha \sin\alpha & 0\\ \cos\alpha \sin\alpha & \sin^{2}\alpha & 0\\ 0 & 0 & 0 \end{array}\right]$$

${\vec{u}}_{Ki}$ signs the vector of kinematic excitation related to the *i*-th link. Vectors ${\vec{u}}_{K2}$ to ${\vec{u}}_{K4}$ equal to 0. The vector ${\vec{u}}_{K1}$ (17) of link 1 becomes:

(17) $${\vec{u}}_{K1}=\left[\begin{array}{c} u_{K1}\cos\alpha \\ u_{K1}\sin\alpha \\ 0 \end{array}\right]$$

The general planar motion can be described by three differential motion Equations (18)–(20) that reflect linear movement in the direction of *x* and *y* axes and rotary movement around the *z*-axis:

(18) $$\frac{d}{dt}{\left(\frac{\partial E}{\partial {\vec{\dot{u}}}_{L}}\right)}_{x}+{\left(\frac{\partial U}{\partial {\vec{u}}_{L}}\right)}_{x}+{\left(\frac{\partial D}{\partial {\vec{\dot{u}}}_{L}}\right)}_{x}=0$$

(19) $$\frac{d}{dt}{\left(\frac{\partial E}{\partial {\vec{\dot{u}}}_{L}}\right)}_{y}+{\left(\frac{\partial U}{\partial {\vec{u}}_{L}}\right)}_{y}+{\left(\frac{\partial D}{\partial {\vec{\dot{u}}}_{L}}\right)}_{y}=F_{0y}$$

(20) $$\frac{d}{dt}{\left(\frac{\partial E}{\partial \vec{\dot{\varphi }}}\right)}_{z}+{\left(\frac{\partial U}{\partial \vec{\varphi }}\right)}_{z}+{\left(\frac{\partial D}{\partial \vec{\dot{\varphi }}}\right)}_{z}=0$$

**Figure 12:**
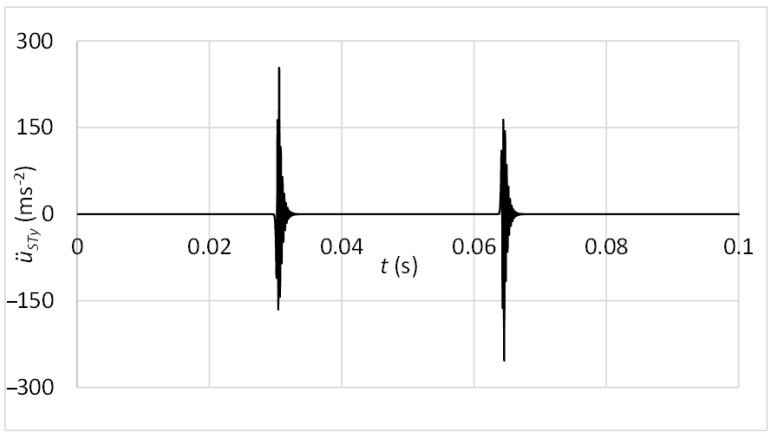
Vibrations at the sensor position—connection with the magnet.

The differential motion equations were processed in MATLAB software. For recognition of vibration motion at the acceleration sensor position, the movement in the *y* direction is demanded. In the case of the standard sensor attachment via the magnet, the acceleration of the diagnostic part in the y direction ${\ddot{u}}_{Ly}$ can be considered as acceleration measured by the sensor at its position, thus, ${\ddot{u}}_{STy}={\ddot{u}}_{Ly}$. The result of vibration acceleration at the sensor position is shown in Figure 12.

**Figure 13:**
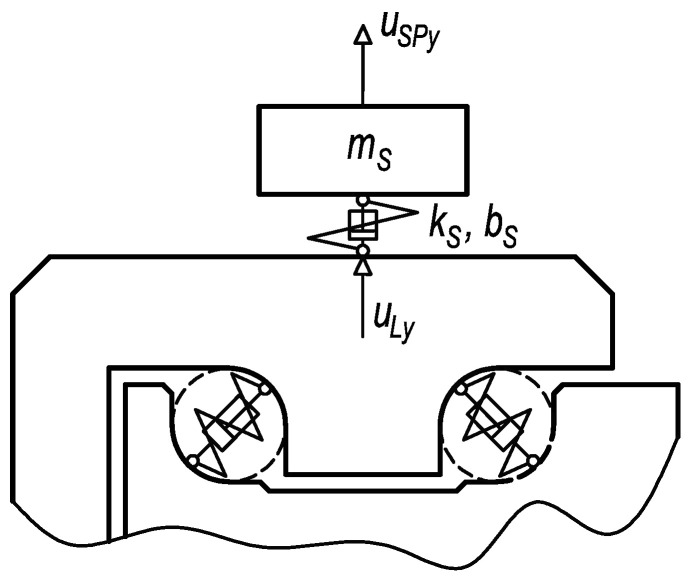
The mechanical model of the sensor connected via rubber spring and additional weight.

In Figure 12, high-frequency vibrations may be recognised. This frequency is related to the natural frequency of the diagnostic part. In a practical case, the natural frequency fluctuates according to various numbers of rolling elements in contact with the diagnostic part raceways. Unfortunately, also, components of vibrations associated with the other frequencies of working mechanisms can be observed in a high-frequency band. Therefore, the innovative attachment of the acceleration sensor was designed. By suitable setting of connection stiffness and mass of additional weight, the frequency of dominant vibrations produced by the damage can be decreased to band out of the operating frequencies. When the acceleration sensor is placed via the rubber spring, the vibration acceleration may be computed in a simplified method as one mass mechanical model (Figure 13).

The dynamic system is due to the damage excited kinematically by the function of the diagnostic part position in the *y* direction at the sensor placement, whereas this function is identical to *u_Ly_*. The damping coefficient *b_S_* (21) approximately equals:

(21) $$b_{S}≅2b_{Srel}\sqrt{k_{S}m_{S}}$$

wherein the damping ratio *b_Srel_* = 0.1 is related to the rubber material [34]. The mass properties and parameters of elastic and damping links are specified in Table 4.

The differential motion Equation (22) reflects the mechanical model in Figure 13.

(22) $$m_{S}{\ddot{u}}_{SPy}+b_{S}\left({\dot{u}}_{SPy}-{\dot{u}}_{Ly}\right)+k_{S}\left(u_{SPy}-u_{Ly}\right)=0$$

**Figure 14:**
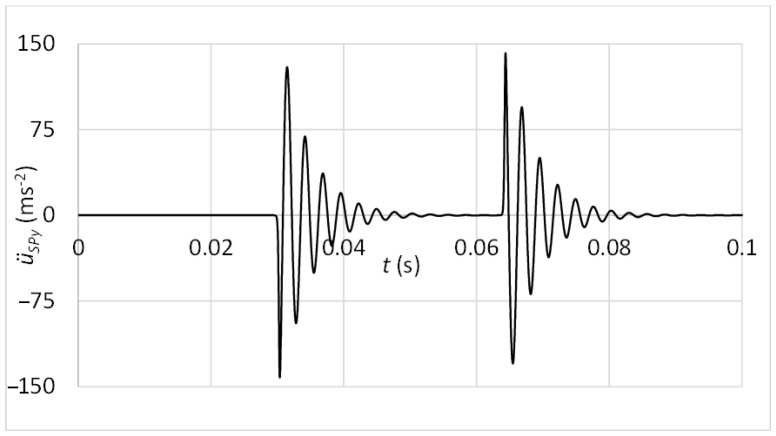
Vibrations at the sensor position—connection with the rubber spring and additional weight.

The differential motion equation was processed utilising MATLAB software. The result of vibration acceleration at the sensor position is shown in Figure 14.

### The Innovative Method

**Figure 15:**
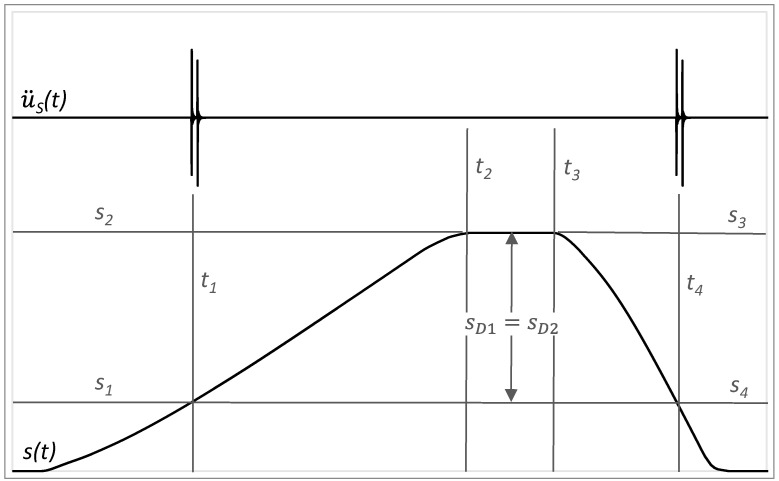
Acceleration amplitudes of vibrations in the context of distance by the damage of guiding profile.

The innovative method of status identification of linear rolling guides is based on evaluation vibrations measured on the diagnostic part in the context of linear rolling guide distance by forward and backward movement. The damage is detected when the increased peaks occur at the identical position. At the limit range of damage to the guiding profile, the increased value of acceleration appears at the distance of *S_D_*_1_ to the end of forward motion and at the distance of *S_D_*_2_ from the beginning of backward motion. From Figure 15, it may be recognised that these distances are equal, so *S_D_*_1_ = *S_D_*_2_.

**Figure 16:**
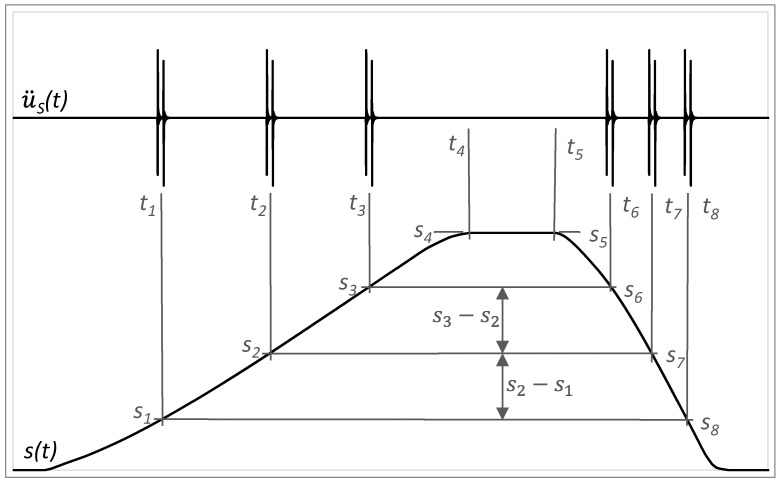
Acceleration amplitudes of vibrations in the context of distance by the damage of rolling elements.

At the limit range of damage to the rolling elements, the increased value of acceleration may appear more times through the motion, which is given by the recirculation process of rolling elements in the carriage. In Figure 16, risen amplitudes appear at the distance of *S*_4_ − *S*_1_, *S*_4_ − *S*_2_ and *S*_4_ − *S*_3_ to the end of forward motion and at the distance of *S*_5_ − *S*_6_, *S*_5_ − *S*_7_ and *S*_5_ − *S*_8_ from the beginning of backward motion. It may be considered that *S*_4_ − *S*_1_ = *S*_5_ − *S*_8_, *S*_4_ − *S*_2_ = *S*_5_ − *S*_7_ and *S*_4_ − *S*_3_ = *S*_5_ − *S*_6_. Also, distances between particular increased amplitudes must be equal by forward or backward motion, so *S*_2_ − *S*_1_ = *S*_3_ − *S*_2_ and *S*_6_ − *S*_7_ = *S*_7_ − *S*_8_.

## 4. Results and Discussion

The innovative method for status identification of linear rolling guides was verified by laboratory testing. At first, the damage to the guiding profile was simulated by grinding the raceway surface, and the connection of the acceleration sensor via magnet and rubber spring was evaluated.

**Figure 17:**
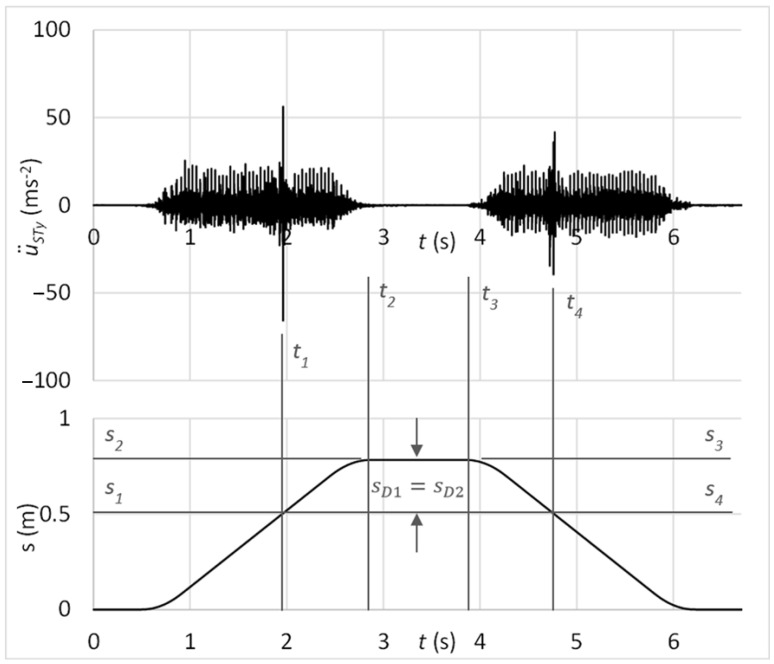
Acceleration of vibrations in the context of distance by the damage of the guiding profile and sensor connection with the magnet.

The time graph in Figure 17 shows the measured acceleration of vibrations on the diagnostic part by the sensor connected with the magnet. In the graph, there might be recognised peaks of vibrations acceleration which may be analysed in the context of carriage position. It can be observed that these peaks occur at the same position of carriage by forward and backward motion, so *S_D_*_1_ = *S_D_*_2_. Therefore, it can be stated that increased peaks of vibration acceleration belong to the simulated damage of the guiding profile.

**Figure 18:**
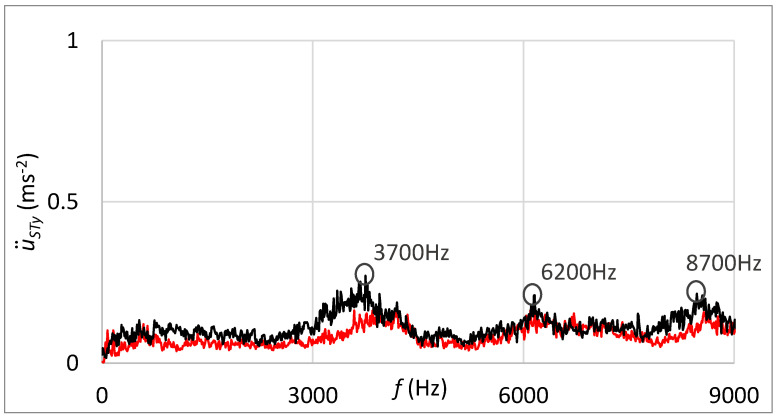
Frequency spectrum of vibrations with the sensor connection by the magnet; black: the damage of the guiding profile; red: without the damage.

Figure 18 shows, in addition, the frequency spectrum of measured vibrations. The black curve is related to the state with damage; the red curve belongs to the state without damage. By comparing these two frequency spectra, the damage cannot be deduced.

**Figure 19:**
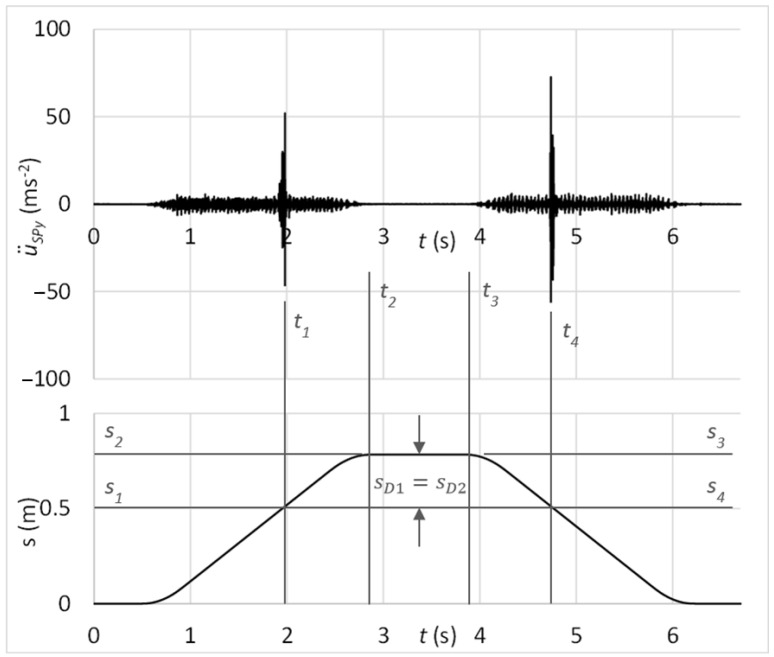
Acceleration of vibrations in the context of distance by the damage of the guiding profile and sensor connection with the rubber spring and additional weight.

The time graph in Figure 19 shows the measured acceleration of vibrations on the diagnostic part by the sensor connected with the rubber spring and additional weight. In the graph, there might be observed peaks of vibration acceleration that belong to the simulated damage of the guiding profile, as *S_D_*_1_ = *S_D_*_2_.

**Figure 20:**
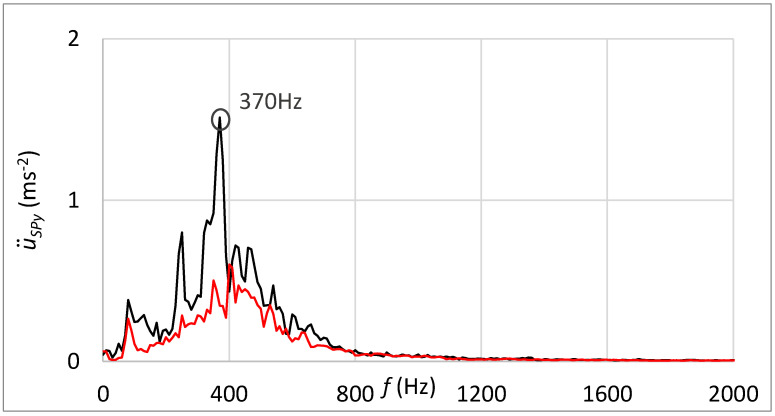
Frequency spectrum of vibrations with the sensor connection by the rubber spring and additional weight; black: the damage of the guiding profile; red: without the damage.

Figure 20 shows the frequency spectrum of measured vibrations by the sensor connected through the rubber spring. The black curve is related to the state with damage; the red curve belongs to the state without damage. By comparing these two frequency spectra, increased vibration amplitude can be noticed at the frequency that can be placed in relation to the damage. The damped high-band operating frequencies can be observed in Figure 20 in contrast with the frequency spectrum shown in Figure 18, which is related to the standard sensor connection.

**Figure 21:**
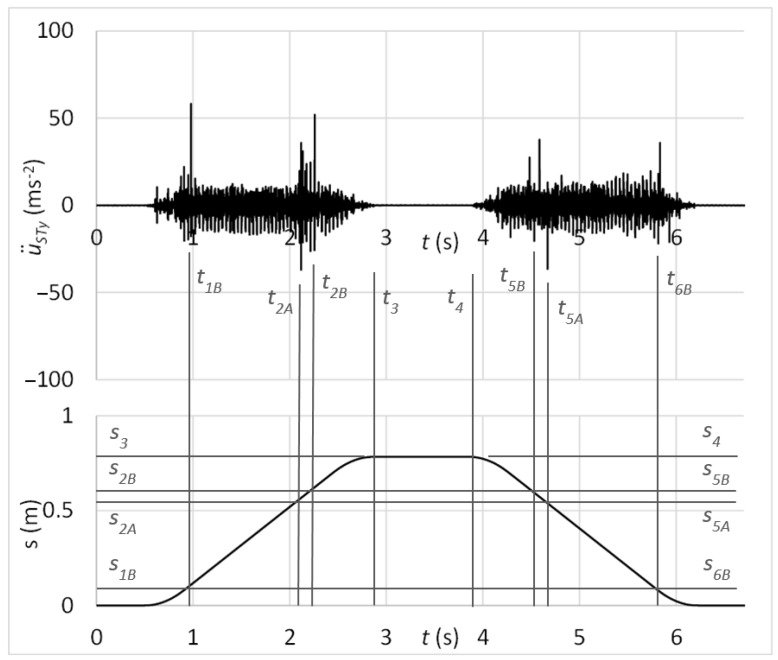
Acceleration of vibrations in the context of distance by the damage of rolling elements and sensor connection with the magnet.

Next, the testing process focused on the verification of the proposed diagnostic method by simulating the rolling elements’ damage. The time graph in Figure 21 shows the measured acceleration of vibrations on the diagnostic part by the sensor connected with the magnet. In the graph, there might be observed peaks of vibration acceleration that belong to the simulated damage of rolling elements, as *S*_3_ − *S*_1*B*_ = *S*_4_ − *S*_6*B*_, *S*_3_ − *S*_2*A*_ = *S*_4_ − *S*_5*A*_ and *S*_3_ − *S*_2*B*_ = *S*_4_ − *S*_5*B*_. It should be noticed that through the time graph of vibration acceleration, it is quite problematic to determine whether the risen peaks belong to the damage. As more increased amplitudes can be observed, it can be decided about the damage only through analysis in the context of carriage position.

**Figure 22:**
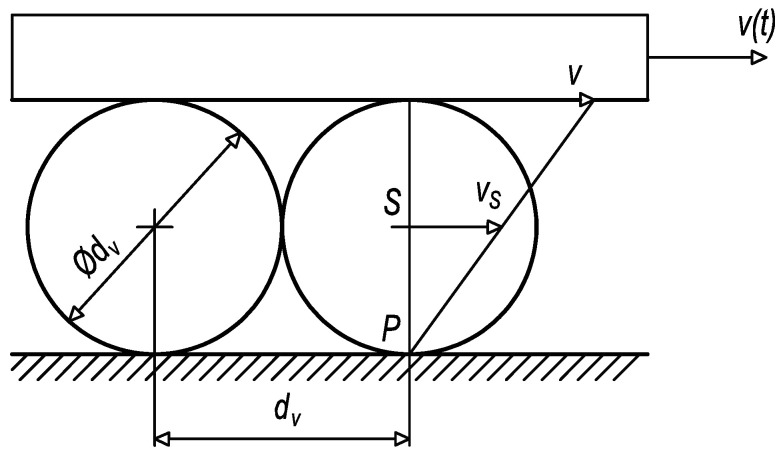
Kinematic scheme of rolling motion.

In addition, it can be proved through kinematic analysis that times, resp. positions between outer peaks relate to the one-turn circulation of damaged rolling elements. From the kinematic scheme in Figure 22, it can be derived the time of one-turn circulation that can be compared with time parameters *t*_1*B*_ = 0.989795 s, *t*_2*B*_ = 2.269760 s, *t*_5*B*_ = 4.541485 s, and *t*_6*B*_ = 5.826715 s in the graph depicted in Figure 21. So, one-turn circulation of the damaged rolling elements by the forward motion (23) equals:

*T_cD_*_1_ = *t*_2*B*_ − *t*_1*B*_ = 1.279965 s (23)

and by the backward motion (24):

*T_cD_*_2_ = *t*_6*B*_ − *t*_5*B*_
*=* 1.285230 s (24)

From the kinematic scheme, the position of carriage (25) can be determined:

*s*(*t*) = *v*(*t*)*t* (25)

Then, the position of the rolling element’s centre (26) is:

*s_S_*(*t*) = *v_S_*(*t*)*t* (26)

By consideration (27) of the relation between the velocity of carriage *v*(*t*) and the velocity of the rolling element’s centre *v_S_*(*t*):

*v_S_*(*t*) = *v*(*t*)/2 (27)

it can be stated that (28):

*s*(*t*) = 2*s_S_*(*t*) (28)

If the carriage contains 32 rolling elements in one circulation raceway, then the distance travelled by one-turn circulation (29) equals:

*S_c_* = 2*S_cS_* = 2 × 32*d_v_* = 512 mm (29)

So, in the case of a constant velocity of *v* = 0.4 ms^−1^, the time of one-turn circulation (30) is:

*T_c_* = *s_c_*/*v* = 1.28 s (30)

By comparing results from Equations (23), (24), and (30), it can be stated that the damage to rolling elements occurs twice during one forward or backward movement.

**Figure 23:**
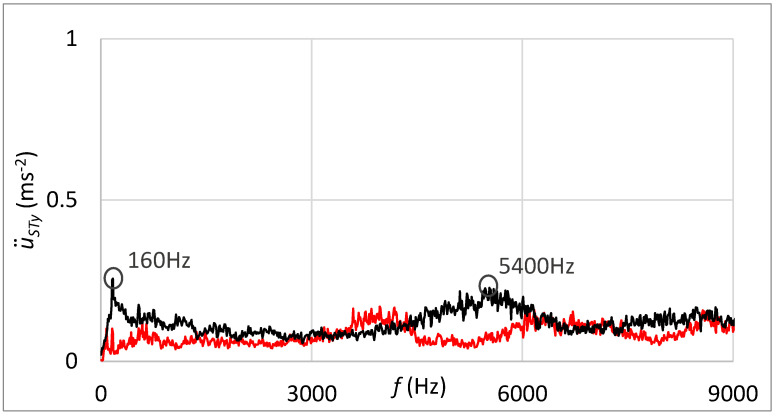
Frequency spectrum of vibrations with the sensor connection by the magnet; black—the damage of rolling elements; red—without the damage.

The frequency spectrum of measured vibrations shown in Figure 23 supplements results processed in the time domain. The black curve is related to the state with damage; the red curve belongs to the state without damage. By comparing these two frequency spectra, the damage cannot be deduced.

**Figure 24:**
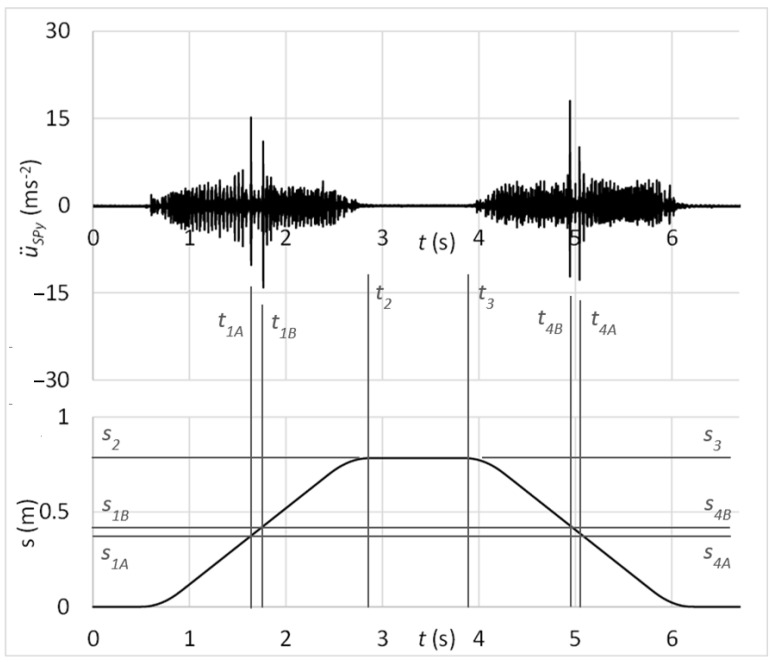
Acceleration of vibrations in the context of distance by the damage of rolling elements and sensor connection with the rubber spring and additional weight.

The time graph in Figure 24 shows the measured acceleration of vibrations on the diagnostic part by the sensor connected with the rubber spring and additional weight. In the graph, there might be observed peaks of vibration acceleration that belong to the simulated damage of rolling elements, as *S*_2_ − *S*_1*B*_ = *S*_3_ − *S*_4*B*_ and *S*_2_ − *S*_1*A*_ = *S*_3_ − *S*_4*A*_.

**Figure 25:**
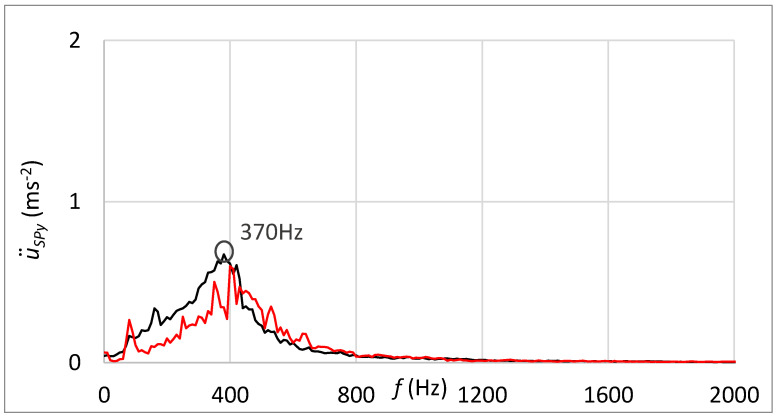
Frequency spectrum of vibrations with the sensor connection by the rubber spring and additional weight; black—the damage of rolling elements; red—without the damage.

Figure 25 shows the frequency spectrum of measured vibrations. The black curve is related to the state with damage; the red curve belongs to the state without damage. By comparing these two frequency spectrums, the damage cannot be deduced. However, the damped high-band operating frequencies can be observed in Figure 25 in contrast with the frequency spectrum shown in Figure 23, which is related to the standard sensor connection.

**Figure 26:**
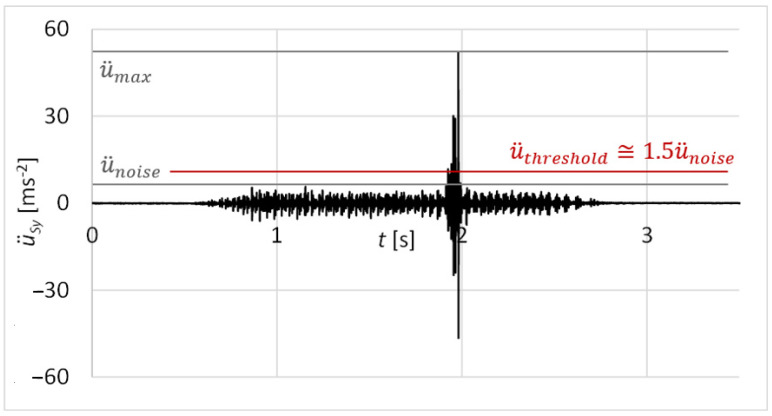
Threshold value of vibration acceleration.

The results of the measurements were statistically evaluated as the reliability of damage identification by the proposed diagnostic method at one forward and backward movement of the functional sample. The measured values of vibration acceleration were analysed as a continuous normal random variable. The evaluated velocity was the maximum amplitude of vibration acceleration ${\ddot{u}}_{max}$ related to the damage (Figure 26). Statistical file numbered *n* = 30 forward and backward movements of the functional sample.

The probability of damage identification at just one forward and backward movement can be solved through two interdependent phenomena, *A*1 and *A*2.

Phenomenon *A*1: At the forward motion, the maximum value of acceleration amplitude ${\ddot{u}}_{max}$ is higher than a threshold ${\ddot{u}}_{threshold}$; ${\ddot{u}}_{max}$ > ${\ddot{u}}_{threshold}$.

Phenomenon *A*2: At the backward motion, the maximum value of acceleration amplitude ${\ddot{u}}_{max}$ is higher than the threshold ${\ddot{u}}_{threshold}$; ${\ddot{u}}_{max}$ > ${\ddot{u}}_{threshold}$.

The merging of two interdependent phenomena represents their intersection. The probability of phenomenon *A* (31), the damage is identified just at one forward and backward movement, equals:

*P*(*A*) = *P*(*A*1 × *A*2) (31)

The probability of phenomena *A*1 and *A*2 (32), (33) can be computed utilising the standard normal random variable *u_t_* and its distribution function *ϕ*(*u_t_*). The probability of phenomena *A*1 and *A*2 is:

*P*(*A*1) = 1 − *ϕ*(*u_tA_*_1_) (32)

*P*(*A*2) = 1 − *ϕ*(*u_tA_*_1_) (33)

The value of the standard normal random variable *u_t_* (34) referring to the threshold of vibration acceleration ${\ddot{u}}_{threshold}$ is then:

(34) $$u_{t}=\left({\ddot{u}}_{threshold}-{\ddot{u}}_{m}\right)/\sigma_{\ddot{u}}$$

wherein ${\ddot{u}}_{m}$ signs a mean value (35) of maximum acceleration amplitudes:

(35) $${\ddot{u}}_{m}=\frac{1}{n}\overset{n}{\underset{i=1}{\sum }}{\ddot{u}}_{maxi}$$

and $\sigma_{\ddot{u}}$ means a standard deviation (36) of maximum acceleration amplitudes.

(36) $$\sigma_{\ddot{u}}=\sqrt{Var_{\ddot{u}}}$$

A variance of maximum acceleration amplitudes $Var_{\ddot{u}}$ (37) can be calculated as:

(37) $$Var_{\ddot{u}}=\frac{1}{n}\overset{n}{\underset{i=1}{\sum }}{\left({\ddot{u}}_{maxi}-{\ddot{u}}_{m}\right)}^{2}$$

The results of the statistical evaluation are summarised in Table 5 and Table 6. In Table 5, there can be recognised statistical values for the guiding profile damage identification. Table 6 summarises statistical values for the rolling elements’ damage identification. Both tables show results by columns for sensor connection by magnet and rubber spring in the case of *A*1 phenomenon (forward movement) and *A*2 phenomenon (backward movement). In tables, the probabilities *P*(*A*1) and *P*(*A*2) of *A*1 and *A*2 phenomena can be found, as well as the probability of damage identification *P*(*A*), which is identical to the reliability of damage identification.

The laboratory testing proved the proposed diagnostic method of damage assessment of linear rolling guides. Results of tests showed the capability of the method for early identification of the guiding profile or rolling elements damage, even if the linear rolling guide was operated under external loads. From time graphs of vibration acceleration, shown in Figure 17, Figure 19, Figure 21, and Figure 24, in the context of the carriage position, the damage can be easily stated due to the obviously risen amplitudes of acceleration.

Statistical evaluation of obtained results showed suitability for involving the innovative principle of acceleration sensor connection through the rubber spring. Due to the damping of high-band operating frequencies, the innovative connection improved the reliability of damage identification from 66% to 98% at the guiding profile damage and from 71% to 100% in the case of rolling elements damage. Damping of high-band operating frequencies is noticeable by comparing frequency spectra of vibration acceleration at the standard sensor connection by the magnet (Figure 18 and Figure 23) and at the unique sensor connection by rubber spring (Figure 20 and Figure 25). In addition, the damage can be noticeable in the frequency spectrum as the increased peak at the specific frequency (Figure 20) if the innovative connection of the sensor is utilised. However, the frequency spectrum cannot be decisive for damage identification.

Results of the research showed that the innovative method, due to the high reliability obtained, could be applicable in industrial practice. Therefore, the next research should focus on the design of the carriage with the integrated diagnostic part, e.g., in the shape of a modular design with or without the diagnostics. In the context of the design, the dynamic and static capacity of the linear rolling guide must be defined, as well as its service life. Also, the autonomous algorithm for the damage assessment should be developed according to the proposed innovative method.

## 5. Conclusions

The diagnostics of linear rolling guides can be solved through the original principle described in the patent [25], especially in the case of great external loads when other diagnostic principles fail. The original principle is based on integrating the load-free diagnostic part into the linear rolling guide carriage. The diagnostic part shares the guiding profile and rolling elements with the carriage and contains the vibration sensor.

This principle was used for the innovative method proposal of damage assessment to linear rolling guides. The method consists of the evaluation of measured vibration acceleration on the diagnostic part. Values of acceleration in the time domain are placed into context with the position of the linear rolling guide carriage. If the increased peak of vibration acceleration appears in the same carriage position at forward and backward movement, the damage to the guiding profile or rolling elements can be stated.

The proposed innovative method was improved by utilising the unique attachment of the acceleration sensor to the diagnostic part. The specific frequency related to the damage could be defined utilising the rubber spring with the additional weight. In addition, the high-band operating frequencies were damped by decreasing this specific frequency. Thus, greater reliability of the proposed diagnostic method was reached.

Laboratory testing realised on the functional sample of the diagnostics proved the suitability of the proposed method for early identification of the damage, even if the linear rolling guide is operated under substantial external loads. In combination with the unique sensor connection, the reliability obtained in laboratory conditions was 98% for identifying the guiding profile damage. In the case of the rolling elements damage, the reliability in laboratory conditions was 100%. The reliability of the innovative diagnostic method was computed by statistical evaluation of data measured on the functional sample of the proposed diagnostics at 30 forward and backward movements for each particular damage condition.

The main benefits of the proposed innovative diagnostic method are:

- High reliability of the diagnostic system for early damage identification to the guiding profile or rolling elements;
- The diagnostic system is independent of linear rolling guides operating conditions, e.g., external loads, linear motion characteristics, and dynamic behaviour of the machine.

## Figures and Tables

**Table 1:** Parameters of Bosch Rexroth linear rolling guide.

Type	Basic Dynamic Capacity	Rolling Elements	Diameter of Rolling Element
KWD 45 FNS	*C* = 86.4 kN	Balls	*d_v_* = 8 mm

**Table 2:** Mass properties.

Mass of the Diagnostic Part	Moment of Inertia to the *x*-Axis	Moment of Inertia to the *y*-Axis	Moment of Inertia to the *z*-Axis	Position of Center of Gravity in the *x*-Axis
*m* = 0.15 kg	*J_x_* = 1.96 × 10^−4^ kg.m^2^	*J_y_* = 1.96 × 10^−4^ kg.m^2^	*J_z_* = 1.96 × 10^−4^ kg.m^2^	*x_T_* = −1.85 × 10^−2^ m

**Table 3:** Positions and parameters of elastic and damping links.

Position of Elastic and Damping Links in the *x*-Axis	Position of Elastic and Damping Links in the *y*-Axis	Angle of Elastic and Damping Link	Stiffness of Elastic Links	Damping Coefficient
*χ* = 23 mm	*ψ* = 4 mm	*α* = 50°	*k* = 1.1 × 10^8^ N.m^−1^	*b* ≅ 800 Ns.m^−1^

**Table 4:** Mass properties and parameters of elastic and damping links.

Mass of the Sensor and Additional Weight	Stiffness of Elastic Links	Damping Coefficient
*m_S_* = 0.02 kg	*k_S_* = 1.1 × 10^5^ N.m^−1^	*b_S_* ≅ 10 Ns.m^−1^

**Table 5:** Reliability of guiding profile damage identification.

		Connection by Magnet		Connection by Rubber Spring	
		*A*1	*A*2	*A*1	*A*2
${\ddot{u}}_{treshold}$		33.75 ms^−2^		10.50 ms^−2^	
${\ddot{u}}_{m}$		47.70 ms^−2^	56.10 ms^−2^	45.98 ms^−2^	38.62 ms^−2^
$\sigma_{\ddot{u}}$		19.56 ms^−2^	19.66 ms^−2^	17.66 ms^−2^	12.51 ms^−2^
*P*(*A*1)	*P*(*A*2)	0.76	0.87	0.98	0.99
*P*(*A*)		0.66		0.97	

**Table 6:** Reliability of rolling elements damage identification.

		Connection by Magnet		Connection by Rubber Spring	
		*A*1	*A*2	*A*1	*A*2
${\ddot{u}}_{treshold}$		28.50 ms^−2^		10.50 ms^−2^	
${\ddot{u}}_{m}$		42.13 ms^−2^	35.14 ms^−2^	14.51 ms^−2^	18.87 ms^−2^
$\sigma_{\ddot{u}}$		14.77 ms^−2^	6.06 ms^−2^	0.45 ms^−2^	1.02 ms^−2^
*P*(*A*1)	*P*(*A*2)	0.82	0.86	1	1
*P*(*A*)		0.71		1	

## Data Availability

Due to the copyright and license rights to the developed diagnostic system that are related to the granted European patent EP3702632B1, we cannot share the technical details of the described solution. However, in the case of interest, we can provide the technical details based on an individual request.
